# Resibufogenin Targets the ATP1A1 Signaling Cascade to Induce G2/M Phase Arrest and Inhibit Invasion in Glioma

**DOI:** 10.3389/fphar.2022.855626

**Published:** 2022-05-17

**Authors:** Xun Zhang, Zhong Yao, Zhiyi Xue, Shuai Wang, Xuemeng Liu, Yaotian Hu, Yan Zhang, Jian Wang, Xingang Li, Anjing Chen

**Affiliations:** ^1^ Department of Neurosurgery, Qilu Hospital, Cheeloo College of Medicine and Institute of Brain and Brain-Inspired Science, Shandong University, Jinan, China; ^2^ Shandong Key Laboratory of Brain Function Remodeling and Jinan Microecological Biomedicine Shandong Laboratory, Jinan, China; ^3^ Hillman Cancer Center, University of Pittsburgh Medical Center, Pittsburgh, PA, United States; ^4^ Department of Biomedicine, University of Bergen, Bergen, Norway

**Keywords:** resibufogenin, GBM, proliferation, invasion, ATP1A1

## Abstract

Resibufogenin (RB) is a major active ingredient in the traditional Chinese medicine Chansu and has garnered considerable attention for its efficacy in the treatment of cancer. However, the anticancer effects and underlying mechanisms of RB on glioblastoma (GBM) remain unknown. Here, we found that RB induced G2/M phase arrest and inhibited invasion in a primary GBM cell line, P3#GBM, and two GBM cell lines, U251 and A172. Subsequently, we demonstrated that RB-induced G2/M phase arrest occurred through downregulation of CDC25C and upregulation of p21, which was caused by activation of the MAPK/ERK pathway, and that RB inhibited GBM invasion by elevating intercellular Ca^2+^ to suppress the Src/FAK/Paxillin focal adhesion pathway. Intriguingly, we confirmed that upon RB binding to ATP1A1, Na^+^-K^+^-ATPase was activated as a receptor and then triggered the intracellular MAPK/ERK pathway and Ca^2+^-mediated Src/FAK/Paxillin focal adhesion pathway, which led to G2/M phase arrest and inhibited the invasion of GBM cells. Taken together, our findings reveal the antitumor mechanism of RB by targeting the ATP1A1 signaling cascade and two key signaling pathways and highlight the potential of RB as a new class of promising anticancer agents.

## 1 Introduction

Glioblastoma multiforme (GBM, WHO IV) accounts for nearly 51.4% of all kinds of brain cancer and remains the leading cause of cancer-related death due to intracranial malignant disease (Preusser et al., 2011). Currently, the standard of therapy for newly diagnosed patients is maximum safe resection followed by adjuvant ionizing radiotherapy and temozolomide chemotherapy (Stupp et al., 2005). However, median survival has remained stagnant over the last decade, remaining at 14.6 months (Hegi et al., 2005; Linz 2010; Stupp et al., 2014). Developing novel anticancer strategies in addition to chemotherapy, radiation and immunotherapy will be critical for patients with glioma. Therefore, low-toxicity, effective drugs to treat glioma and prolong the survival of patients are urgently needed.

Natural ingredient extracts have historically been an important resource for potential cancer therapies. Chansu, a dried secretion of *Bufo gargarizan*s Cantor or *B. melanostictus Schneider*, is a widely used traditional Chinese medicine that exhibits a variety of pharmacological activities, including anti-inflammatory, detoxifying, analgesic, stimulatory, and anticancer properties (Sun et al., 2016; Apryani et al., 2020; Song et al., 2021). Chansu was reported to significantly inhibit the malignant progression of glioma (Lan et al., 2018), lung cancer (Yu et al., 2014) and breast cancer (Dong et al., 2011). Resibufogenin (RB), alternatively known as bufogenin and recibufogenin, is a major active ingredient in cinobufacini. RB has been commonly used for the treatment of a variety of tumors in recent years. For instance, RB was demonstrated to inhibit TAK1-mediated NF-κB activity through protein kinase C-dependent repression of GSK-3 in pancreatic cancer (Liu et al., 2018). Moreover, RB inhibited the malignant progression of ovarian clear cell carcinoma by suppressing the PI3K/AKT and actin cytoskeleton signaling pathways. Nevertheless, the effect and the molecular mechanisms of RB on glioma are currently unclear.

The dysregulated cell cycle of glioma cells is the main cause of their malignant proliferation and is a classic therapeutic target for oncology drug development (Thomas et al., 2014). The cell G2/M phase transition is mediated by the activation of the CDK1-Cyclin B complex (Chang and Ferrell 2013). Activation of CDK1 is mediated by dephosphorylation of CDK1 at Tyr15 by CDC25C (Tsuchiya, Murai and Yamashita 2015). Additionally, the elevated expression of p21 can downregulate the expression of CDK1-Cyclin B complex to induce G2/M phase arrest (Chang et al., 2000). It has been demonstrated that signaling mediated by mitogen-activated protein kinases (MAPKs) plays an important role in regulating the cell cycle and responding to DNA damage (Schaeffer and Weber 1999). MAPKs are classified into three subfamilies based on their sequence similarity and the nature of their upstream activators: JNK/SAPK, ERK1/2 and p38. Extracellular signal-regulated kinase (ERK) is a major transmitter of extracellular signals that connect cellular membrane receptor stimulation to alterations in cellular function (Robinson and Cobb 1997; Roux and Blenis 2004; Ramos 2008). The transient activation of ERK is critical in cell proliferation, whereas continuous ERK activation triggers cell G2/M phase arrest by promoting CDC25C ubiquitination and proteasomal degradation (Eymin et al., 2006; Kar et al., 2006; Guo et al., 2011; Wang et al., 2016) and by upregulating p21 expression (Karkhanis and Park 2015). Additionally, the newly developed clinical drug Ibrance, the first CDK4/6 inhibitor approved by the FDA in 2015, targets the cell cycle and may be effective and safe against certain types of breast cancer (Thomas et al., 2014).

In addition to the uncontrollable proliferation of glioma, invasion is another distinctive feature of its malignant phenotype. The focal adhesion complex composed of integrins serves as the primary site of cell adhesion to the extracellular matrix and interacts with the actin cytoskeleton to regulate cell motility. The dynamic regulation of focal adhesion and the associated actin cytoskeleton reorganization are critical factors for cell invasion (Carragher and Frame 2004). Focal adhesion kinase (FAK) was discovered to associate with Src via a Src homology domain-mediated interaction after it was initially identified as a tyrosine phosphorylated protein in cells transformed by the Src oncogene (Kanner et al., 1990; Schaller et al., 1992; Cobb et al., 1994). The FAK-Src complex interacts with and phosphorylates a number of adaptor proteins, including P130cas (Hsia et al., 2005) and Paxillin (Schlaepfer, Mitra and Ilic 2004), to promote tumor invasion. Furthermore, the ever-present second messenger Ca^2+^ has been identified as a vital regulator of cancer cell invasion by regulating the dynamic cycle of focal adhesion complex assembly and disassembly (Chang et al., 2017; Wu et al., 2021). Exploring RB’s target proteins in glioma cells will help us better understand RB’s anticancer mechanisms and generate new treatment options for glioma. Chansu has been reported to inhibit Na^+^-K^+^-ATPase (Bick et al., 2002; Wang, Sun and Heinbockel 2014). Na^+^-K^+^-ATPase, which is found in the plasma membrane, is a classic ion transporter enzyme that exchanges Na^+^ for K^+^ in cells. ATP1A1 is one of 4 isoforms of the Na^+^-K^+^-ATPase alpha subunit identified in humans (Clausen, et al., 2017). When Na^+^-K^+^-ATPase is only partially inhibited, there is no significant disruption of the homeostasis of intracellular Na^+^ and K^+^. Depending on the cell after activation by the appropriate ligand, Na^+^-K^+^-ATPase stimulates the proliferation of healthy cells (Aydemir-Koksoy, Abramowitz and Allen 2001; Chen et al., 2019) or, in contrast, inhibits the proliferation of tumor cells (Prassas et al., 2011). Moreover, ATP1A1 can activate the MAPK/ERK pathway (Ono et al., 2016), and Na^+^-K^+^-ATPase can alter intracellular Ca^2+^ concentrations (Bick et al., 2002). Therefore, exploring the mechanisms of RB in glioma will identify new clinical agents for the targeted treatment of glioma.

Taken together, our study shows that when RB binds to ATP1A1, Na^+^-K^+^-ATPase is activated as a receptor, accompanied by activation of the intracellular MAPK/ERK pathway and inhibition of the Ca^2+^-regulated Src/FAK/Paxillin focal adhesion pathway, after which GBM cells undergo G2/M phase arrest and inhibition of invasion.

## 2 Materials and Methods

All animal procedures were approved by the Ethics Committee of Qilu Hospital of Shandong University (permit number: DWLL-2-21-042). This study was conducted in full adherence to relevant regulations and guidelines.

### 2.1 Cell Lines and Culture

The human GBM cell lines U251 and A172 were purchased from the Chinese Academy of Sciences Cell Bank (Shanghai, China). Normal human astrocyte (NHA) cells were kindly provided by Prof. Rolf Bjerkvig at the Department of Biomedicine, University of Bergen, Norway. Complete medium was used to maintain the GBM cell lines and NHA cells: Dulbecco’s modified Eagle’s medium (DMEM; Thermo Fisher Scientific; Waltham, MA, United States) supplemented with 10% fetal bovine serum (FBS; Thermo Fisher Scientific), streptomycin (100 g/ml), and penicillin (100 U/mL). Prof. Rolf Bjerkvig of the Department of Biomedicine at the University of Bergen, Norway kindly provided the patient-derived GBM stem-like cell (GSC) line P3#GBM. P3#GBM was obtained from Haukeland University Hospital (Bergen, Norway) following approval of the local ethics committee (approval number 2009/117) (Golebiewska et al., 2020). In addition, P3#GBM primary cells have been cultured in Prof. Rolf Bjerkvig’s laboratory since 2009, and several associated articles have been published (Wang et al., 2009; Han et al., 2020). P3#GBM cells were cultured in serum-free Neurobasal™ medium (Gibco/Thermo Fisher Scientific) supplemented with 2% B-27 Neuro Mix (Thermo Fisher Scientific), 20 ng/ml epidermal growth factor (EGF; Thermo Fisher Scientific), 10 ng/ml basic fibroblast growth factor (bFGF; PeproTech; Rocky Hill, NJ, United States), and streptomycin (100 g/ml), and the cells were incubated at 37°C in a humidified atmosphere composed of 95% air and 5% CO_2_.

### 2.2 Chemical and Reagents

RB (purity ≥99.69%, CAS# 465-39-4), purchased from MedChemExpress (MCE; Monmouth, NJ, United States), was dissolved in dimethyl sulfoxide (DMSO; Sigma–Aldrich; St. Louis, MO, United States) and stored at −20°C as a stock solution (10 and 50 mM). Ruthenium red (RR; purity ≥95.0%, CAS# 11,103-72-3) purchased from MCE was dissolved in DMSO and stored at −20°C as a 10 mM stock solution. U0126 (CAS# 109,511-58-2) purchased from Beyotime (Shanghai, China) was dissolved in DMSO as a 10 mM stock solution and stored at −20°C. The stock solutions were diluted directly in complete medium, and the final DMSO concentration was <0.1%.

The following antibodies were used: MMP-2 (#87809S, CST; Boston, MA, United States), Vimentin (#5741S, CST), N-cadherin (#13116S, CST), Snail (#3879S, CST), phospho-ERK (p-ERK,Thr202/Tyr204, #4370S, CST), ERK (#4695S, CST), phosphor-JNK (p-JNK,Thr183/Tyr185, #4668S, CST), JNK (#9552S, CST), phospho-p38 (p-p38, Thr180/Tyr182, #4511S, CST), p38 (#8690S, CST), GAPDH (#5174S, CST), p21 (#2947S, CST), CDC25C (#4688S, CST), phospho-CDK1 (p-CDK1, Tyr15, #9111S, CST), CDK1 (#28439S, CST), Cyclin B1 (#12231S, CST), Src (#2109S, CST), phosphor-Src (p-Src, Ser17, #12432SS, CST), ATP1A1 (#23565S, CST) Cyclin B2 (#ab185622, Abcam; Cambridge, MA, United States), FAK (#ab40794, Abcam), phospho-FAK (p-FAK, Y397, #ab81298, Abcam), Paxillin (#ab32084, Abcam), and phospho-Paxillin (p-Paxillin, Y118, #ab109547, Abcam). Secondary antibodies against goat anti-rabbit/mouse were purchased from Zhongshan Golden Bridge Biotechnology (Zhongshan Golden Bridge; Beijing, China). The luminance was determined using a chemiluminescence imager (Bio–Rad ChemiDoc XRS+; Hercules, CA, United States) in accordance with the manufacturer’s protocol.

### 2.3 Cell Viability Assay

The Cell Counting Kit-8 assay (CCK-8; Dojindo, Kumamoto, Japan) was performed to determine cell viability. To obtain an accurate IC_50_ of each cell, we experimented with different concentration gradients. For P3#GBM cells, we used a concentration gradient of RB at 0, 0.5, 1, 1.5, 2, 2.5, 3 and 3.5 μM for 48 h. For the GBM cell lines U251 and A172, we used RB concentrations of 0, 1.5, 3, 4.5, 6, 7.5 and 9 μM for 48 h. For NHA cells, we used a concentration gradient of RB at 0, 5, 15, 20, 25, 30, 35, 40 and 45 μM for 48 h. All final DMSO concentrations were <0.1%. P3#GBM, U251 and A172 cells (4 × 10^3^ cells/well) were inoculated in 96-well plates and incubated at 37°C. After 24 h, the original culture medium was replaced with 100 μL of culture medium containing various doses of RB or vehicle control (diluted DMSO). After 24, 48 and 72 h of treatment, P3#GBM, U251 and A172 cells were incubated in 10 μL of CCK-8 reagent with 90 μL of serum-free DMEM for 1 h. An EnSight multimode plate scanner was used to detect the absorbance at 450 nm (PerkinElmer, Hopkinton, MA, United States).

### 2.4 Colony Formation Assay

U251 and A172 cells (800 cells/well) were inoculated in 6-well plates. After cell attachment, U251 and A172 cells were treated with DMSO or RB (2 or 4 μM), and then fresh medium was replaced once every 3 days for the duration of the expression. After incubation for 14 days, 4% paraformaldehyde was used to fix U251 and A172 colonies for 5 min, and then 0.5% crystal violet was used to stain U251 and A172 colonies for 15 min and washed three times with PBS. A bright-field microscope was used to count colonies with more than 50 cells (Leica; Solms, Germany).

### 2.5 Cell Proliferation Assay

5-ethynyl-2′-deoxyuridine (EdU) binding assays were used to detect the binding of EdU, a thymidine analog, to proliferating cells via a catalytic reaction between EdU and Apollo fluorescent dye (RiboBio; Guangzhou, China). The nuclei of the cells were counterstained with 4′,6-diamidino-2-phenylindole (DAPI). The number of EdU-positive cells in each well was determined using a fluorescence microscope (Leica, Solms, Germany).

### 2.6 Western Blot

Western blotting was performed as previously described (Yao et al., 2020). P3#GBM, U251 and A172 cells were treated with RB for 48 h and then lysed in RIPA lysis buffer (Beyotime) with protease and phosphatase inhibitors (Beyotime) for 30 min and sonicated to enhance lysis. The luminescence intensity was detected with a chemiluminescence imager (Bio–Rad ChemiDoc XRS+; Hercules, CA, United States).

### 2.7 Cell Cycle Assays

P3#GBM, U251 and A172 (4 × 10^5^) were inoculated in 6-well plates. After treating cells with (diluted DMSO) or RB (2 and 4 μM) for 48 h, the cells were digested, centrifuged, and incubated overnight at 4°C in cold 70% ethanol. Samples were centrifuged, stained with propidium iodide (BD Biosciences; San Jose, CA, United States) for 10-15 min protected from light and subjected to cell cycle analysis by flow cytometry (Accuri C6, BD Biosciences). ModFit software (Becton Dickinson; San Diego, CA, United States) was used to identify P3#GBM, U251 and A172 cell cycle distributions.

### 2.8 Invasion Assays

To determine how RB affected GBM cell invasion, we carried out three invasion assays: transwell invasion assays, 3D spheroid invasion assays and GBM-brain organoid coculture invasion assays.

The transwell invasion assay was performed using 24-well Matrigel invasion chambers (Costar, Cambridge, MA, United States). Briefly, Matrigel was mixed with serum-free cell culture medium at 4°C in a 1:8 dilution ratio. One hundred microliters were applied uniformly to the surface of the polycarbonate membrane in the upper chamber and left at 37°C for 1 h to polymerize into a gel. The cells were suspended in inserts (2 × 10^4^ cells/200 μL). DMEM containing 30% fetal bovine serum was added to the lower chamber (600 μL). U251 and A172 cells were treated with vehicle control (diluted DMSO) or RB (2 or 4 μM) for 48 h. Fixation, staining, digital imaging and counting of cells under invading membranes.

In the 3D spheroid invasion assay, P3#GBM, U251 or A172 cells (3,000 cells/well) were inoculated in a U-shaped 96-well plate (Trevigen; Gaithersburg, MD, United States) to develop tumor spheroids for 3 days, and then tumor spheroids were embedded in invasive matrix (Trevigen; Gaithersburg, MD, United States). Medium containing vector control (diluted DMSO) or RB (2 or 4 μM) was replaced with fresh complete medium, and 0-h spheroids were pictured as a reference point to measure the invasive area. The relative invasion area of tumor spheroids was measured on 6^th^ day.

Our previous work described the protocol for culturing 18-days rat fetal brain organoids for the GBM brain organoid coculture invasion *ex vivo* system (Bjerkvig, Laerum and Mella 1986). After 3 weeks in culture, the differentiation of cells in normal brain organoids was complete, and brain organoids could be exposed to tumor organoids. U251-GFP and A172-GFP cells (n = 3,000), established using GFP lentiviruses, were plated in 96-well plates for 3 days to generate tumor spheroids and then cocultured with mature brain organoids for 24 h. GBM organoids were treated with diluted DMSO or 4 μM RB for 0, 48 and 96 h. Confocal microscopy captured coculture images of tumor cell invasion (Leica DMi8; Solms, Germany). ImageJ (National Institutes of Health; Bethesda, United States) software was used to analyze the GBM cell-related invasive area.

### 2.9 Na^+^-K^+^-ATPase Activity Determination

Na^+^-K^+^-ATPase activities were determined by a Na^+^-K^+^-ATPase kit (A070-2-2; Nanjing Jiancheng Bioengineering Institute; Nanjing, China). The sample and mixed reagents were added according to the manufacturer’s instructions and reacted precisely at 37°C for 10 min. Then, R4 was added and centrifuged for 10 min at 3,500 rpm, and the supernatant was collected. Then, the color developer was added to the supernatant at room temperature for 2 min, and R6 was added for 5 min at room temperature. An EnSight multimode plate scanner was used to detect the absorbance at 636 nm and 1 cm optical diameter (PerkinElmer, Hopkinton, MA, United States).

### 2.10 ROS Measurement

A total of 2–4 ×10^5^ GBM cells were inoculated in each 6-well plate, and the cells were treated with diluted DMSO or 4 μM RB for 48 h. After 48 h of treatment, the cells were washed three times with PBS and incubated with 10 μM 2′,7′-dichlorofluorescein diacetate (DCFH-DA, Sigma–Aldrich) for 30 min. After incubation, DCFH-DA was replaced with PBS. The fluorescence intensity of DCFH-DA was detected by flow cytometry (BD C6, San Jose, CA, United States). FlowJo software was used to analyze flow cytometry data (Treestar; Ashland, OR, United States), and at least 1 × 10^5^ cells were analyzed.

### 2.11 Whole Transcriptome Sequencing

RNA sequencing (LC-Bio; Shanghai, China) was performed on RB-treated P3#GBM cells (4 μM RB) and U251 cells (4 μM RB) and their parent cell lines. Full data accompanying this experiment are available in the Sequence Read Archive (SRA). The accession number is PRJNA796785.

### 2.12 Ca^2+^ Measurement

Fluo-4/AM (MCE) was added to the cells after they had been treated with diluted DMSO or RB (2 or 4 μM) for 48 h. The solution was kept in the dark at room temperature for 30 min. Heated Hank’s balanced salt solution was used to wash U251 and A172 cells. Intracellular Ca^2+^ levels in GBM cells were detected by measuring the fluorescence intensity of the calcium indicator Fluo-4/AM with fluorescence microscopy and flow cytometry. FlowJo software was used to analyze flow cytometry data.

### 2.13 RNA Interference

Short interfering RNA sequences (siRNAs) silencing ATP1A1 (GenePharma Gene; Shanghai, China) were transfected into cells with Lipofectamine 3,000 reagent (Thermo Fisher Scientific). The silencing efficiency in U251 and A172 cells was assessed by immunoblotting 48 h after transfection. The siRNA sequence was as follows: siATP1A1#3 5′-GGA​GGC​UUC​UUU​ACU​UAC​UTT-3′.

### 2.14 Orthotopic Xenograft Model and Bioluminescence Imaging

We constructed P3#GBM cells infected with lentivirus expressing fluorescein-GFP for animal studies. A total of 4 × 10^5^ fluorescein-GFP P3#GBM cells in a 10 μl cell suspension were stereotaxically inoculated into 4-week-old BALB/c nude male mouse brains (1 mm posterior to bregma, 2 mm right of midline suture, depth 1.5 mm) (Han et al., 2019; Johannessen et al., 2019). Male mice were chosen because of the uneven tumor formation in the animal model due to individual differences in sex hormones in female mice. On the 7^th^ day, after determining tumor size, the mice were randomly divided into two groups: the NC (n = 10) and RB groups (n = 10). Mice were administered daily intraperitoneal injections of NC (DMSO) or RB (10 mg/kg/day). Bioluminescence imaging was used to evaluate the size of the tumor every week (IVIS Spectrum, Hopkinton, MA, United States). During imaging, mice were intraperitoneally administered D-fluorescein and potassium salt D (150 mg/kg; Yeasen Biotech; Ltd., Shanghai, China) under isoflurane gas anesthesia. The survival of each mouse was recorded after death. Kaplan–Meier analysis was performed on the median survival of the tumor-bearing mice, and log-rank analysis was used to measure the statistical significance of the differences. Finally, the tumors were cut up and frozen in liquid nitrogen or placed in formalin for immunohistochemistry.

### 2.15 Immunohistochemistry

Tumors were separated from the sacrificed mice, fixed in 4% paraformaldehyde and embedded in paraffin. The paraffin-embedded samples were sectioned (4 μm) and secured on glass slides. The sections were heated in a microwave oven at pH 7.2 in 10 mM citric acid buffer for epitope retrieval. The slides were then incubated with primary antibodies (rabbit anti-Ki-67 1:200 dilution; rabbit anti-ATP1A1 1:100 dilution) overnight at 4°C, followed by incubation with HRP-conjugated secondary antibodies for 1 h at room temperature. Antibodies were detected using the substrate diaminobenzidine (Beyotime), and slides were counterstained with hematoxylin (Beyotime).

Two pathologists independently evaluated each immunostained section under light microscopy. Immunoreactive scores were calculated by multiplying the intensity of staining (from 0 to 4; 0 = negative, 1 = weakly positive; light yellow, 2 = moderately positive; yellowish-brown, 3 = strongly positive; brown) by the percentages of stained-positive cells (from 0 to 100).

### 2.16 Statistical Analysis

At least three biological replicates of each experiment were performed. Data are provided as the mean ± SEM. Statistical analysis was performed using GraphPad Prism 8. The following *p* values were considered significant: ∗*p* < 0.05; ∗∗*p* < 0.01; ∗∗∗*p* < 0.001.

## 3 Results

### 3.1 RB Inhibits GBM Cell Proliferation and Induces GBM Cell Cycle Arrest in the G2/M Phase

**FIGURE 1:**
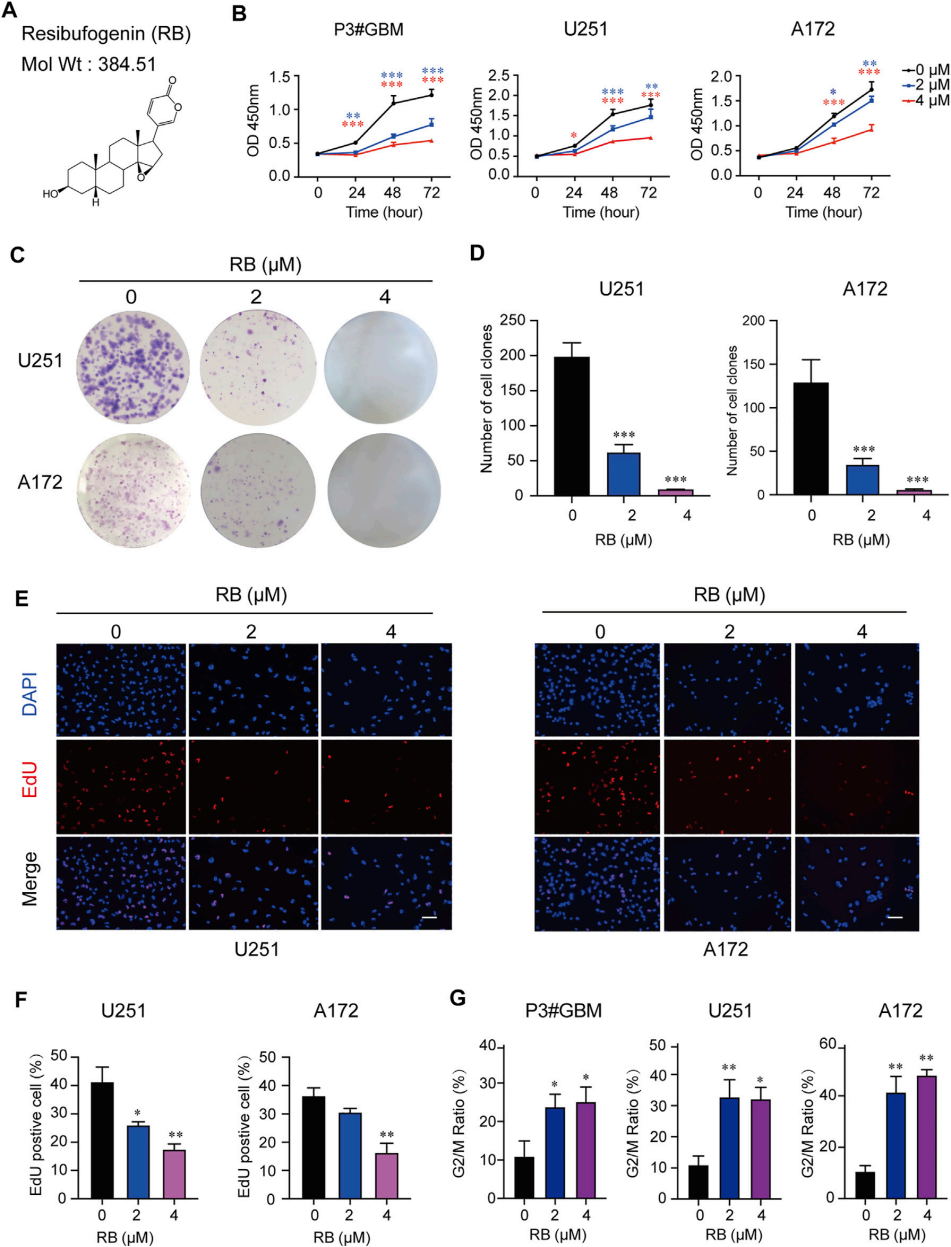
RB inhibits GBM cell proliferation and induces GBM cell cycle arrest in the G2/M phase. **(A)** Chemical structure of RB drawn through ChemDraw 20.0. The molecular weight of RB is 384.51 g/mol. The topological polar surface area of RB is 59.06. P3#GBM, U251 and A172 GBM cells were treated with 2 μM or 4 μM RB under normal culture conditions. **(B)** Using the CCK-8 assay, proliferation curves of P3#GBM, U251 and A172 GBM cells treated with vehicle, DMSO (0), 2 μM or 4 μM RB for 0, 24, 48 or 72 h. **(C)** Representative images of the cell clone formation assay for U251 and A172 cells given DMSO (0), 2 μM or 4 μM RB. **(D)** Graphic representation of the number of colonies shown in **(C)**. **(E)** EdU assays conducted on U251 and A172 cells treated with vehicle, DMSO (0) and 2 or 4 μM RB for 48 h. EdU was detected by Apollo 567 staining (red), nuclei were highlighted by DAPI staining (blue), and images were merged. Scale bar, 50 μm. **(F)** Graphic representation of the percentages of EdU-positive U251 and A172 cells shown in **(E)**. **(G)** Graphic representation of the percentages of G2/M phase cells calculated from each group (Supplementary Figure S1B). Three independent experiments were performed with data shown as the mean ± SEM by two-way ANOVA **(B)** or one-way ANOVA **(D,F,G)**. *p < 0.05; **p < 0.01 and ***p < 0.001.

The chemical structure of RB is shown in Figure 1A. The half-maximal inhibitory concentration (IC_50_) values were 2.29, 3.05 and 6.21 µM in the primary GBM cell line P3#GBM and in two GBM cell lines, U251 and A172. However, the IC_50_ value was 32.66 µM in NHA cells. NHA cells showed proliferation inhibition only at higher concentrations compared to GBM cells, suggesting that RB may be more sensitive to inhibiting GBM cell proliferation at certain concentrations. To determine the cytotoxic effects and inhibitory activity of RB in GBM cells, we treated P3#GBM, U251 and A172 cells with RB at 2 or 4 µM concentrations for 24, 48 and 72 h *in vitro*. CCK-8 assays showed that RB significantly inhibited the proliferation of GBM cells in a concentration-dependent manner (Figure 1B and Supplementary Figure S1A).

To determine whether RB inhibited the proliferation of GBM cells, we performed a colony formation assay. The results showed that RB at 2 μM significantly reduced the number of U251 and A172 cell colonies, and no colonies appeared under 4 μM RB treatment (Figures 1C,D). These results were also confirmed in EdU assays, where the percentages of EdU-positive cells were significantly reduced in the U251 and A172 cells treated with 2 and 4 μM RB (Figures 1E,F), indicating that RB significantly inhibited the proliferation of human GBM cells.

To further determine whether proliferation inhibition induced by RB was associated with cell cycle arrest, we examined the effect of RB on the cell cycle of P3#GBM, U251 and A172 cells. Flow cytometry data indicated that RB significantly inhibited cell cycle progression in P3#GBM, U251 and A172 cells. A significant increase in the G2/M phase fraction was coupled with a decrease in the G0/G1 phase fraction (Figure 1G and Supplementary Figure S1B). Taken together, these results revealed that RB inhibited cell proliferation and induced G2/M-phase cell cycle arrest in GBM cells.

### 3.2 RB Suppresses the Invasion of GBM Cells

**FIGURE 2:**
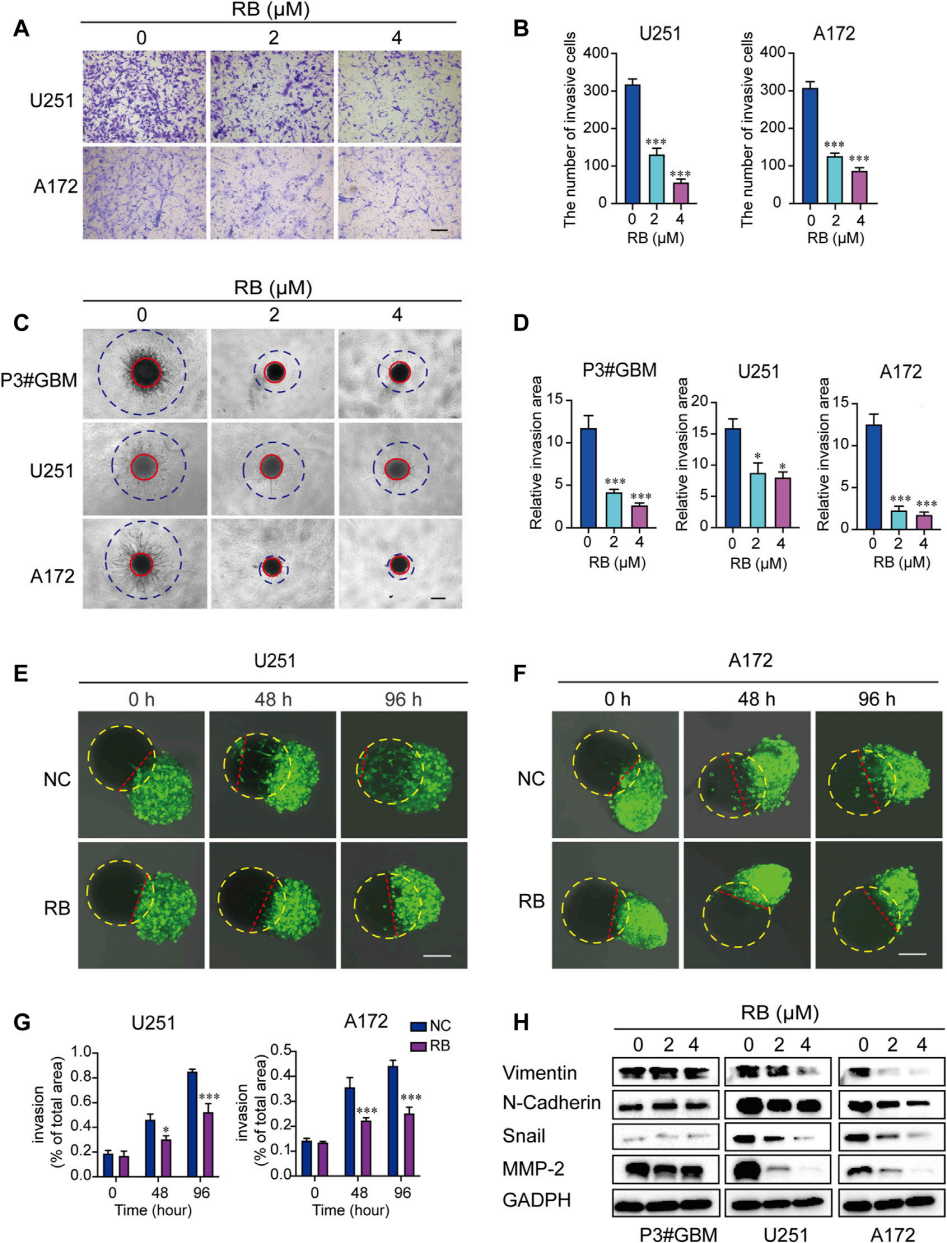
RB inhibits the invasion of GBM cells. **(A)** Representative images of transwell invasion assays. Scale bar, 100 μm. **(B)** The numbers of invasive GBM cells shown in **(A)**. **(C)** Representative images of the 3D spheroid invasion assay performed on P3#GBM, U251 and A172 cells treated with vehicle control (diluted DMSO) or RB (2 or 4 μM) and evaluated on the 6^th^ day. Scale bar, 200 μm. **(D)** Graphic representation of the relative invasive area shown in **(C)**. **(E)** Representative images of coculture invasion assays for U251 cells treated with vehicle, DMSO (0) and 4 μM RB and evaluated at 0, 48, and 96 h. The yellow dotted line represents brain organoids, and the red dotted line represents the U251 cell invasion range. Scale bar, 100 μm. **(F)** Representative images of coculture invasion assays for A172 cells treated with vehicle, DMSO (0) and 4 μM RB and evaluated at 0, 48, and 96 h. The yellow dotted line represents brain organoids, and the red dotted line represents the A172 cell invasion range. Scale bar, 100 μm. **(G)** Quantification of the invasive area after 0, 48, and 96 h of 4 μM RB treatment shown in **(E,F)**. **(H)** Western blot analysis of MMP2 and EMT-related proteins after 4 μM RB treatment for 48 h. Three independent experiments were performed with data shown as the mean ± SEM by two-way ANOVA **(G)** or one-way ANOVA **(B,D)**. *p < 0.05; **p < 0.01, and ***p < 0.001.

**FIGURE 3:**
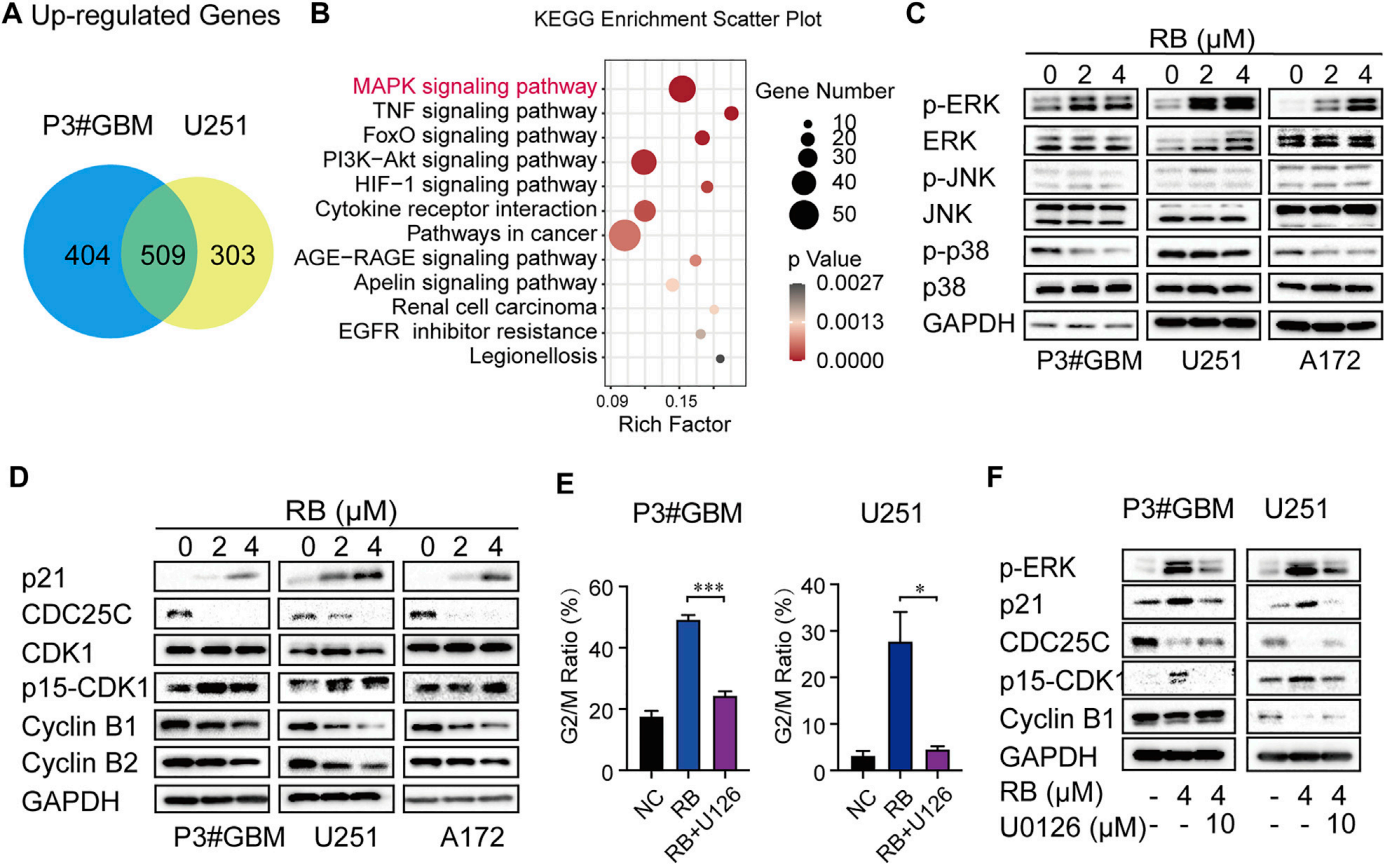
Activation of the ERK/MAPK pathway involves RB-induced G2/M arrest in GBM cells. **(A)** Intersections of genes with upregulated expression in P3#GBM and U251 cells treated with 4 μM RB compared with control cells. **(B)** KEGG pathway enrichment analysis of intersecting genes with upregulated expression. **(C)** Western blot analysis of total and phosphorylated protein levels of ERK, JNK and p38 after 2 or 4 μM RB treatment for 48 h in P3#GBM, U251 and A172 cells compared with control cells. **(D)** G2/M phase-related protein expression after RB treatment for 48 h in P3#GBM, U251 and A172 cells analyzed by western blot. **(E)** P3#GBM and U251 cells were pretreated with U0126 (10 μM; ERK inhibitor) for 6 h, and then, 4 μM RB was incubated for another 48 h. Graphic representation of percentages of G2/M phase cells calculated from each group were shown in Supplementary Figure S1C. **(F)** Western blot analysis of the protein expression of p-ERK, p21, CDC25C, p15-CDK1 and cyclin B1 in P3#GBM and U251 cells pretreated for 6 h with U0126 (10 μM) and then treated with 4 μM RB. Data from three independent experiments were displayed as the mean ± SEM by one-way ANOVA **(E)**. *p < 0.05; **p < 0.01, and ***p < 0.001.

To investigate whether RB affects cell invasion, we performed transwell assays and 3D spheroid-based invasion assays. The results showed that in both transwell (Figures 2A,B) and 3D spheroid-based invasion assays (Figures 2C,D), treatment with RB significantly reduced GBM cell invasion by ∼2–4-fold compared with that in the control cells. Next, to further demonstrate the inhibitory effect of RB on GBM cell invasion, we conducted coculture experiments with GBM spheroid and brain organoid coculture assays. GBM cells were cultured for 3 days to develop tumor spheroids, which were then cocultured with mature rat brain organoids and treated with diluted DMSO or RB (4 μM). As shown in Figures 2E,G, after 48 and 96 h of treatment, the invasive area by GBM spheroids was decreased significantly in the 4 μM RB-treated U251 spheroids compared to the control U251 spheroids. We found the same results in 4 μM RB-treated A172 spheroids, where RB also reduced the area invaded by tumor cells after 48 and 96 h (Figures 2F,G).

In addition, we examined the effect of RB on the protein expression levels of several mesenchymal markers in GBM cells. We found that RB inhibited GBM cell invasion by decreasing the expression of Vimentin, N-cadherin, Snail and MMP-2 (Figure 2H). These data suggested that RB suppressed the invasion of GBM cells.

### 3.3 Activation of the ERK/MAPK Pathway Involves RB-Induced G2/M Arrest in GBM Cells

As shown in Figure 1G and Supplementary Figure S1B, flow cytometry analysis indicated that G2/M phase arrest occurred in GBM cells following 2 or 4 μM RB treatment. To elucidate the mechanisms of RB-induced G2/M arrest in GBM cells, we performed whole transcriptome sequencing in vehicle control (diluted DMSO) and 4 μM RB-treated P3#GBM and U251 cells. The differentially expressed mRNAs were chosen using the cutoffs of *p* value <0.05 and log_2_ fold change >1. Figure 3A shows that 913 and 812 mRNAs had upregulated expression in P3#GBM and U251 cells, respectively, and that the number of intersecting genes with upregulated expression between the two GBM cell lines was 509. To shed light on the biological functions of the intersecting genes, we performed KEGG analysis of the intersecting genes with upregulated expression and found that the MAPK pathway was enriched as the top pathway (Figure 3B). In light of the fact that MAPK families have been shown to play a vital role in cycle transition (Zhang and Liu 2002; Wang et al., 2007), we predicted that the activation of the MAPK pathway might be a key determinant of RB-induced G2/M phase arrest. Then, we examined which kinds of MAPKs mediate RB-induced G2/M phase arrest in GBM cells. As shown in Figure 3C, the total amount of ERK1/2, JNK and p38 MAP kinases did not change significantly. The significant activation of ERK1/2 was observed in P3#GBM, U251 and A172 cells, but not JNK and p38. Only activation of ERK was consistent with the sequencing result that MAPK signaling pathway was significantly upregulated.

Activation of the MAPK/ERK pathway has been reported to induce cancer cells to arrest in G2/M phase by regulating the protein expression levels of p21 and CDC25C (Zhang and Liu 2002; Wang et al., 2007). The levels of several G2/M-related proteins in GBM cells treated with RB were then determined using western blotting. Cyclin B1, Cyclin B2, and CDC25C levels were decreased, whereas p21 and Tyr15-phospho-CDK1 levels were increased and total CDK1 remained unchanged (Figure 3D), indicating that G2/M phase arrest induced by RB was associated with altered levels of key checkpoint-related proteins.

We hypothesized that the activation of the MAPK/ERK pathway contributed to RB-mediated G2/M phase arrest. To ascertain the function of ERK in RB-induced G2/M phase arrest, we pretreated P3#GBM and U251 cells for 6 h with the specific inhibitor of ERK, U0126 (10 μM). As shown by flow cytometry, the ratios of cells in the G2/M phase were markedly increased in cultures treated with 4 μM RB compared to the DMSO control, but RB-mediated G2/M phase arrest was significantly rescued by U0126 in P3#GBM and U251 cells (Figure 3E and Supplementary Figure S1C). Next, we examined whether an inhibitor of ERK modulates the expression of key checkpoint proteins in RB-induced G2/M phase arrest. As shown in Figure 3F, the protein expression level of p-ERK was significantly attenuated by treatment with the ERK inhibitor U0126, indicating that the MAPK/ERK pathway was engaged in RB-induced GBM cell G2/M phase arrest. U0126 restored RB-induced inhibition of Cyclin B1, Cyclin B2 and CDC25C expression and blocked Tyr15-phospho-CDK1 and p21 expression in P3#GBM and U251 cells. These results suggested that RB induced GBM cells to arrest in G2/M phase via the MAPK/ERK/CDC25C/p21/CDK1/Cyclin B pathway.

### 3.4 RB Inhibits GBM Cell Invasion Partially by Elevating Intracellular Ca^2+^ Levels to Suppress the Src/FAK/Paxillin Focal Adhesion Pathway

**FIGURE 4:**
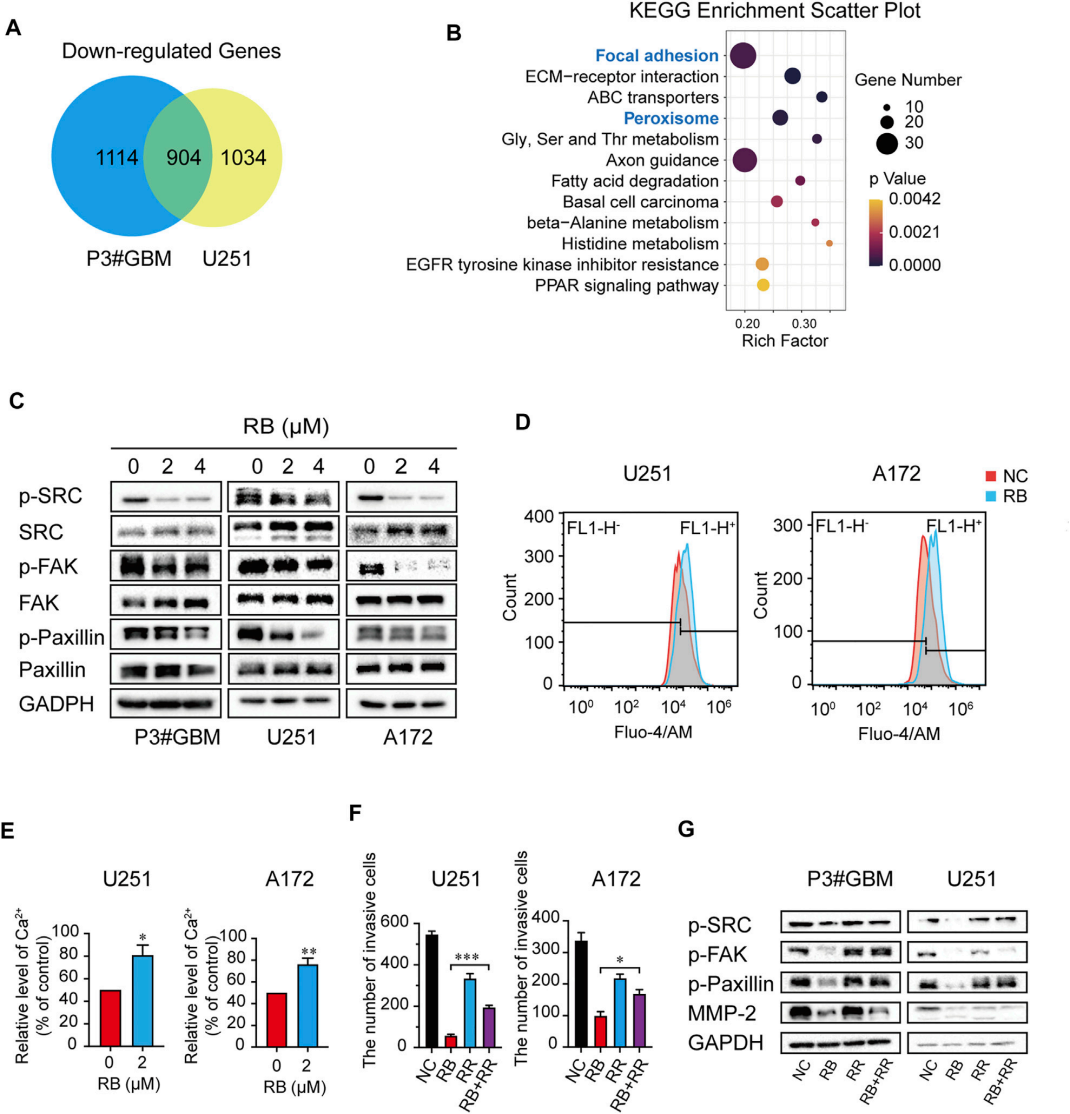
RB inhibits GBM cell invasion partially by elevating intracellular Ca^2+^ levels to suppress the Src/FAK/Paxillin focal adhesion pathway. **(A)** Intersections of genes with downregulated expression in P3#GBM and U251 cells treated with 4 μM RB compared with control cells. **(B)** KEGG pathway enrichment analysis of intersecting genes with downregulated expression. **(C)** P3#GBM, U251 and A172 glioma cells were treated with RB for 48 h. Western blot analysis was used to determine focal adhesion-associated protein expression levels. **(D)** Intracellular Ca^2+^ was measured by Fluo-4/AM with flow cytometry. **(E)** Graphic representation of the mean fluorescence intensity of Fluo-4/AM shown in **(D)**. **(F)** The numbers of invasive GBM cells were shown in Supplementary Figure S2G. **(G)** Western blot analysis showed that blocking Ca^2+^ with RR treatment for 48 h rescued RB-induced inhibition of invasion in U251 and A172 cells. Three independent experiments were performed with data shown as the mean ± SEM by unpaired Student’s t test **(E)** or one-way ANOVA **(F)**. *p < 0.05; **p < 0.01 and ***p < 0.001.

Figure 4A reveals that a total of 2018 and 1938 mRNAs had downregulated expression in P3#GBM and U251 cells, respectively, and that the number of intersecting genes with downregulated expression between the two GBM cell lines was 904. To probe the mechanism of RB-mediated inhibition of GBM cell invasion, we performed KEGG pathway enrichment of intersecting genes with downregulated expression and found that focal adhesion was enriched as the top pathway (Figure 4B).

Src, FAK, and Paxillin are the main elements of focal adhesion, a high-intensity attachment points of cells to the extracellular matrix formed by the leading edge of the invading or attached cells (Stallcup 2017; Schiffer et al., 2018). We hypothesized that RB inhibits GBM cell invasion partly by suppressing the Src/FAK/Paxillin focal adhesion pathway in GBM cells. Our results showed that RB treatment decreased the phospho-Src, FAK, and Paxillin levels, although it had no effect on focal adhesion-related total Src, FAK, and Paxillin levels (Figure 4C), suggesting that RB inhibited GBM cell invasion by suppressing the Src/FAK/Paxillin focal adhesion pathway.

A previous study reported that maintained Ca^2+^ influx can destroy the components of FAK’s focal connections in the presence of Src deficiency, markedly inhibiting cell invasion and motility (Jeong et al., 2020). We further investigated whether RB altered intracellular Ca^2+^ levels to suppress the Src/FAK/Paxillin focal adhesion pathway, thereby inhibiting the invasion of GBM cells.

To determine whether Ca^2+^ in cells was a key target for RB treatment, we first assessed intracellular Ca^2+^ levels using flow cytometry and fluorescence microscopy. As shown in Figures 4D,E and Supplementary Figures S2A,B, intracellular Ca^2+^ levels were significantly elevated in the 4 μM RB-treated cells compared with the control cells. To investigate whether Ca^2+^ elevation was a crucial factor in the RB-inhibited focal adhesion pathway, we first treated RB-treated U251 and A172 cells with RR (3 μM), an L-type calcium current blocker, and decreased Ca^2+^ levels were observed after treatment with the combination of 4 μM RB and 3 μM RR compared to RB treatment alone (Supplementary Figures S2C,D). These same results were also confirmed by flow cytometry, where 3 μM RR blocked the Ca^2+^ elevation induced by RB (Supplementary Figures S2E,F). Furthermore, transwell assays revealed that the combination of 4 μM RB and 3 μM RR significantly rescued invasion in U251 and A172 cells compared to RB treatment alone (Figure 4F and Supplementary Figures S2G), suggesting that elevated Ca^2+^ inhibited invasion in treated cells. Additionally, western blot revealed that the activity of focal adhesion-related proteins inhibited by RB could be rescued by reducing intracellular Ca^2+^ by RR (Figure 4G). Taken together, our results demonstrated that RB inhibited GBM cell invasion partly by elevating intracellular Ca^2+^ levels to suppress the Src/FAK/Paxillin focal adhesion pathway.

### 3.5 RB Inhibits Malignant Progression of GBM by Targeting the ATP1A1 Signaling Cascade and Increasing Na^+^-K^+^-ATPase Activity in GBM Cells

**FIGURE 5:**
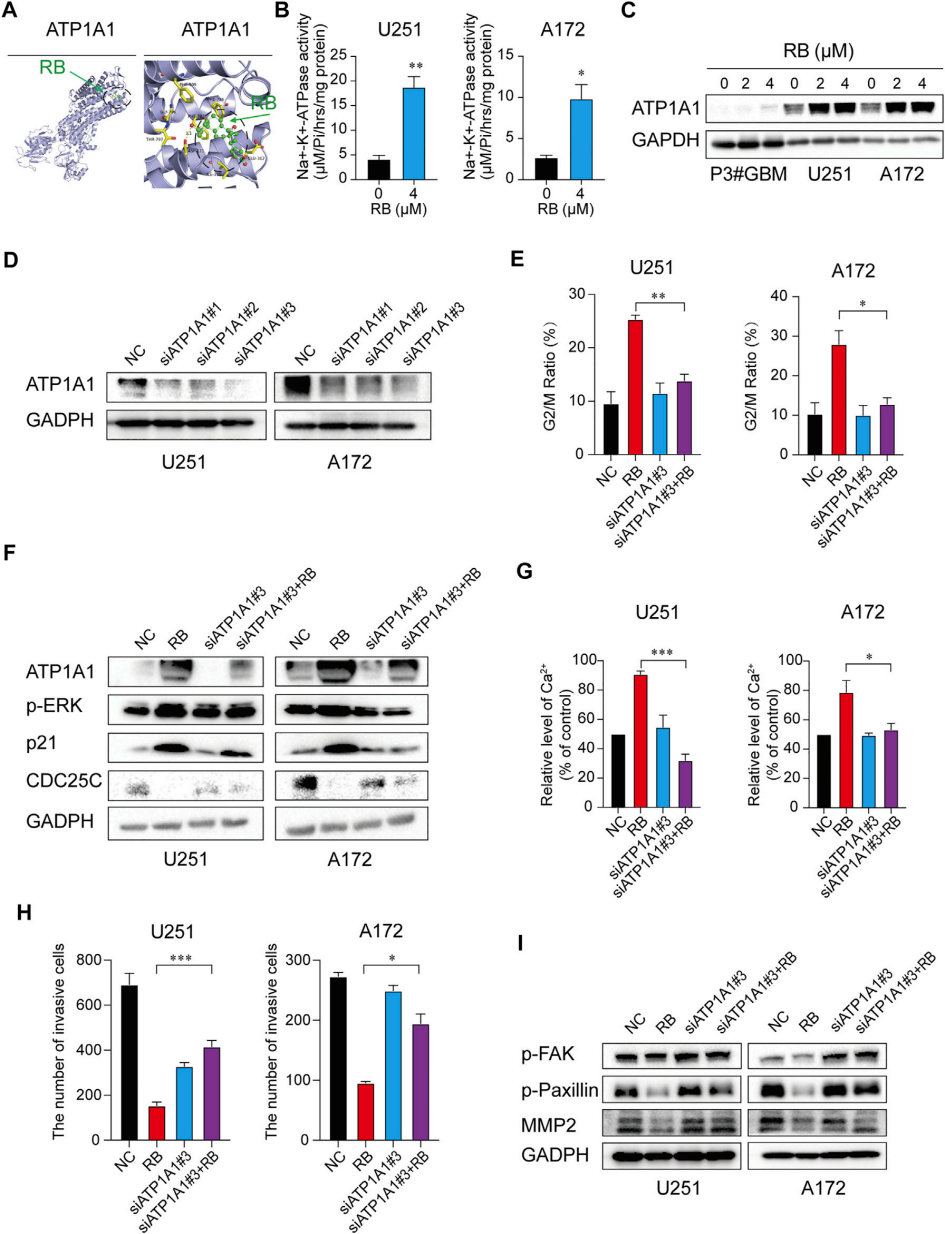
RB inhibits malignant progression of GBM by targeting ATP1A1 and increasing Na^+^-K^+^-ATPase activity in GBM cells. **(A)** The most likely predicted target of RB. The binding energy of RB with the ATP1A1 protein was −8.7 kcal/mol, proving a good binding interaction. RB interacts with the ATP1A1 protein mainly through the formation of hydrogen bonds as well as hydrophobic interactions. The hydrogen bond length with ASP-121 was 3.1 Å; hydrophobic interactions were formed with Ile-315, Glu-312, Phe-785, Phe-783, Phe-909, Gly-796, and Thr-797. **(B)** Graphic representation of Na^+^-K^+^-ATPase activity. **(C)** Representative western blot analysis of total ATP1A1 protein expression. **(D)** Western blotting analysis validated the efficiency of siATP1A1s in GBM cells. **(E)** GBM cells were transfected with siATP1A1#3 for 48 h and incubated with 4 μM RB for 48 h. Graphic representation of the percentages of G2/M phase cells calculated from the NC, RB, siATP1A1#3 and siATP1A1#3 + RB cells (Supplementary Figure S3D). **(F)** Western blot analysis of ATP1A1, p21, CDC25C and G2/M-related proteins from NC, RB, siATP1A1#3 and siATP1A1#3 + RB in U251 and A172 cells. **(G)** Graphic representation of intracellular Ca^2+^ levels from the NC, RB, siATP1A1#3 and siATP1A1#3 + RB groups in U251 and A172 cells (Supplementary Figure S3E). **(H)** The numbers of invasive cells as shown in Supplementary Figure S3F. **(I)** Western blot analysis of the protein expression levels of p-FAK, p-Paxillin and MMP2 in NC, RB, siATP1A1#3 and siATP1A1#3 + RB in U251 and A172 cells. Three independent experiments were performed with data shown as the mean ± SEM by unpaired Student’s t test **(B)** or one-way ANOVA **(E,G,H)**. *, *p* < 0.05; **, *p* < 0.01 and ***, *p* < 0.001.

We found that RB-mediated G2/M phase arrest inhibited GBM cell proliferation by activating the MAPK/ERK pathway, which altered p21/CDC25C/CDK1/Cyclin B protein expression, and RB inhibited GBM invasion by increasing intercellular Ca^2+^ to suppress the Src/FAK/Paxillin focal adhesion pathway. Thus, exploring RB target proteins in GBM cells would provide insight into the mechanisms underlying the inhibition of GBM malignant progression. The traditional Chinese medicine Chansu is known to be a specific inhibitor of Na^+^-K^+^-ATPase. Moreover, previous research showed that RB could alter intracellular K^+^ and Na^+^ concentrations by affecting the function of Na^+^-K^+^-ATPase (Hao et al., 2011). To predict the most likely Na^+^-K^+^-ATPase-related target proteins of RB, we used BioSolveIT SeeSAR software, which indicated that RB may target ATP1A1 in GBM cells (Figure 5A).

First, we tested whether RB regulated Na^+^-K^+^-ATPase enzyme activity in GBM cells and found that treatment with 4 μM RB significantly enhanced Na^+^-K^+^-ATPase activity by ∼4-fold in U251 cells and by ∼6-fold in A172 cells (Figure 5B), indicating that RB significantly increased Na^+^-K^+^-ATPase activity in GBM cells. ROS and Na^+^-K^+^-ATPase develop a feed-forward cycle (Yan et al., 2013; Sodhi et al., 2015; Yan et al., 2017). Activation of Na^+^-K^+^-ATPase could block the Na^+^-K^+^-ATPase/ROS amplification loop as well as increase ATP1A1 expression (Yan et al., 2017). Whole transcriptome sequencing analysis suggested that peroxisomes were inactivated in GBM cells following treatment with RB (Figure 4B and Supplementary Figure S3A). Thus, we detected the levels of intracellular ROS in P3#GBM, U251 and A172 cells treated with RB. As expected, the levels of intracellular ROS were significantly decreased following treatment with RB (Supplementary Figures S3B,C). Furthermore, we tested ATP1A1 expression and found that RB significantly increased the expression of ATP1A1 (Figure 5C). These results showed that activation of Na^+^-K^+^-ATPase with RB upregulated ATP1A1 expression by blocking the Na^+^-K^+^-ATPase/ROS amplification loop. ATP1A1 may be the target by which RB inhibits the malignant progression of GBM.

To further clarify the target of RB in GBM cells, we knocked down ATP1A1 using siRNA in U251 and A172 cells. Our results showed that siATP1A1#3 efficiently knocked down ATP1A1 at the protein level in U251 and A172 cells (Figure 5D). In the cells transfected with ATP1A1 siRNAs, RB treatment markedly alleviated G2/M phase arrest induced by RB alone (Figure 5E and Supplementary Figure S3D). Next, after silencing ATP1A1, RB treatment markedly blocked the protein levels of p21, CDC25C, p-ERK and CDK1 compared to RB treatment alone in U251 and A172 cells (Figure 5F).

Furthermore, in cells transfected with ATP1A1 siRNAs, RB treatment substantially alleviated intracellular Ca^2+^ levels (Figure 5G and Supplementary Figure S3E) and restored the invasiveness of GBM cells (Figure 5H and Supplementary Figure S3F). Furthermore, in U251 and A172 cells transfected with ATP1A1 siRNAs, treatment with RB substantially restored the protein expression of MMP2 and the phosphorylation of FAK and Paxillin compared to RB treatment alone (Figure 5I). These results showed that RB inhibited the malignant progression of GBM by targeting the ATP1A1 signaling cascade and increasing Na^+^-K^+^-ATPase activity in GBM cells.

### 3.6 RB Exerts Antitumor Effects *in vivo*

**FIGURE 6:**
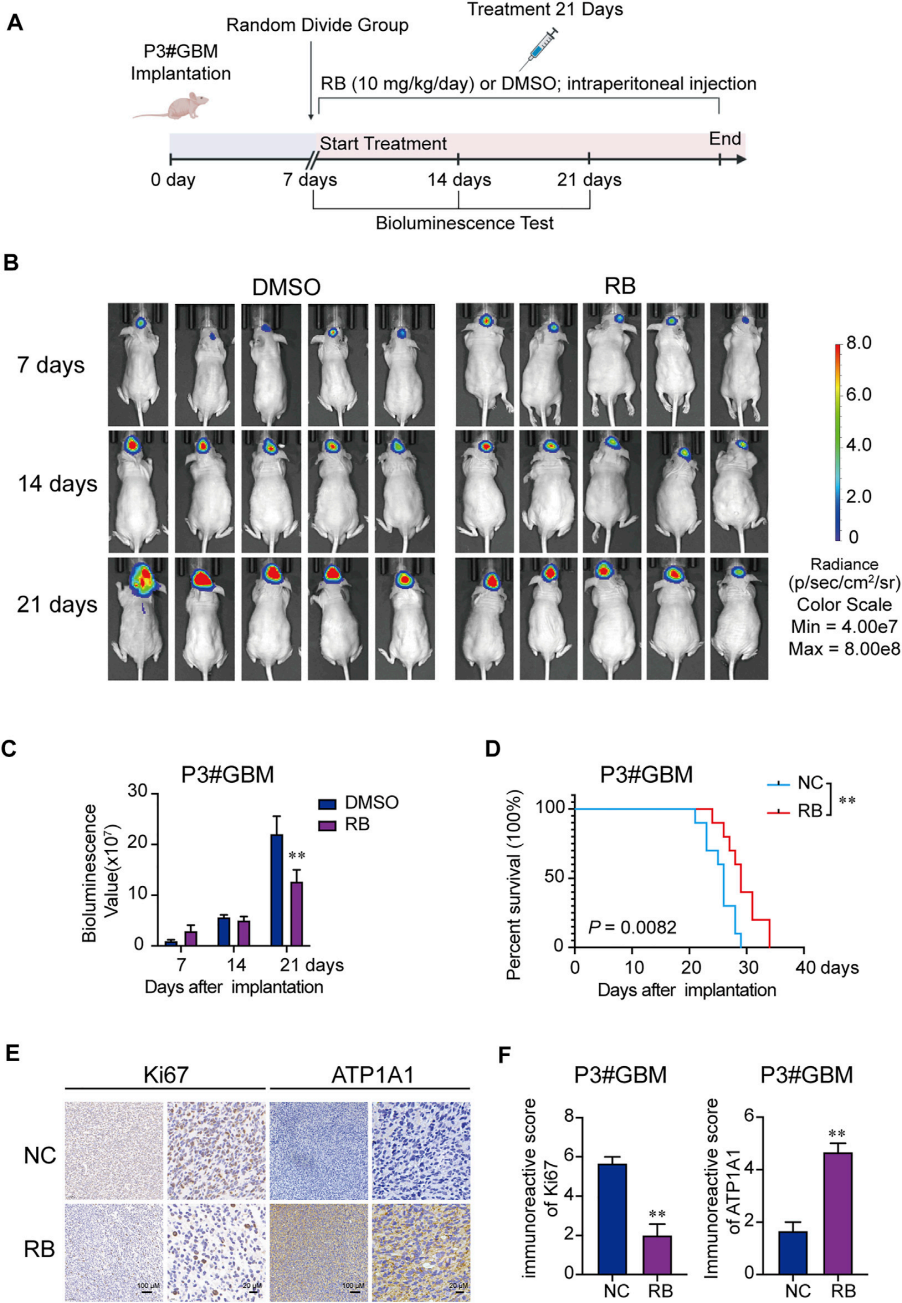
RB exerts antitumor efficacy *in vivo*. **(A)** Scheme of tumor inoculation and systemic injection. **(B)** P3#GBM-luciferase cells were orthotopically inoculated into BALB/c nude mice. PerkinElmer IVIS Spectrum monitored tumor growth by detecting bioluminescence. **(C)** Bioluminescence values on the 7^th^, 14^th^, and 21^st^ days after implantation. **(D)** Kaplan–Meier analysis was performed on the median survival of the tumor-bearing mice, and log-rank analysis was used to measure the statistical significance of the differences. **(E)** Images of IHC staining for ATP1A1 and Ki-67 in sections from brains of orthotopic P3#GBM tumor-bearing nude mice treated with DMSO or RB. Scale bars, 100 and 20 μm. **(F)** Immunoreactive scores of Ki-67 and ATP1A1 in sections from the brains of orthotopic P3#GBM tumor-bearing nude mice treated with DMSO or RB in **(E)**. Three independent experiments were performed with data shown as the mean ± SEM by two-way ANOVA **(C)** or unpaired Student’s t test **(F)**. Survival differences between groups were assessed by the log-rank test **(D).** *p < 0.05; **p < 0.01 and ***p < 0.001.

The efficacy of RB was evaluated using an orthotopic tumor modeling approach from P3#GBM-luciferase-expressing cells inoculated in BALB/c nude mice bregma. As shown in Figure 6A, after the 7^th^ day of implantation, mice were selected randomly into two groups (n = 10/group): the NC (DMSO) and RB groups (10 mg/kg/day), and tumor growth was evaluated using luciferase bioluminescence. The bioluminescence values of the two groups of mice showed a significant difference by 21 days (Figure 6B). On the 21st day after implantation, bioluminescence values were nearly 42% lower in the RB-treated group than in the DMSO-treated group (Figure 6C). Kaplan–Meier analysis was performed on the median survival of the tumor-bearing mice, and log-rank analysis was used to measure the statistical significance of the differences. As shown in Figure 6D, RB prolonged the median survival of the tumor-bearing mice from 26 to 29 days (*p* = 0.0082). We also investigated the expression of Ki-67 and ATP1A1 by immunohistochemical (IHC) staining. The expression of Ki-67 was markedly lower under RB treatment, suggesting that RB suppresses the proliferation of GBM *in vivo* (Figures 6E,F). The expression of ATP1A1 was increased after RB treatment, suggesting that RB inhibited the growth of GBM by targeting Na^+^-K^+^-ATPase.

## 4 Discussion

**FIGURE 7:**
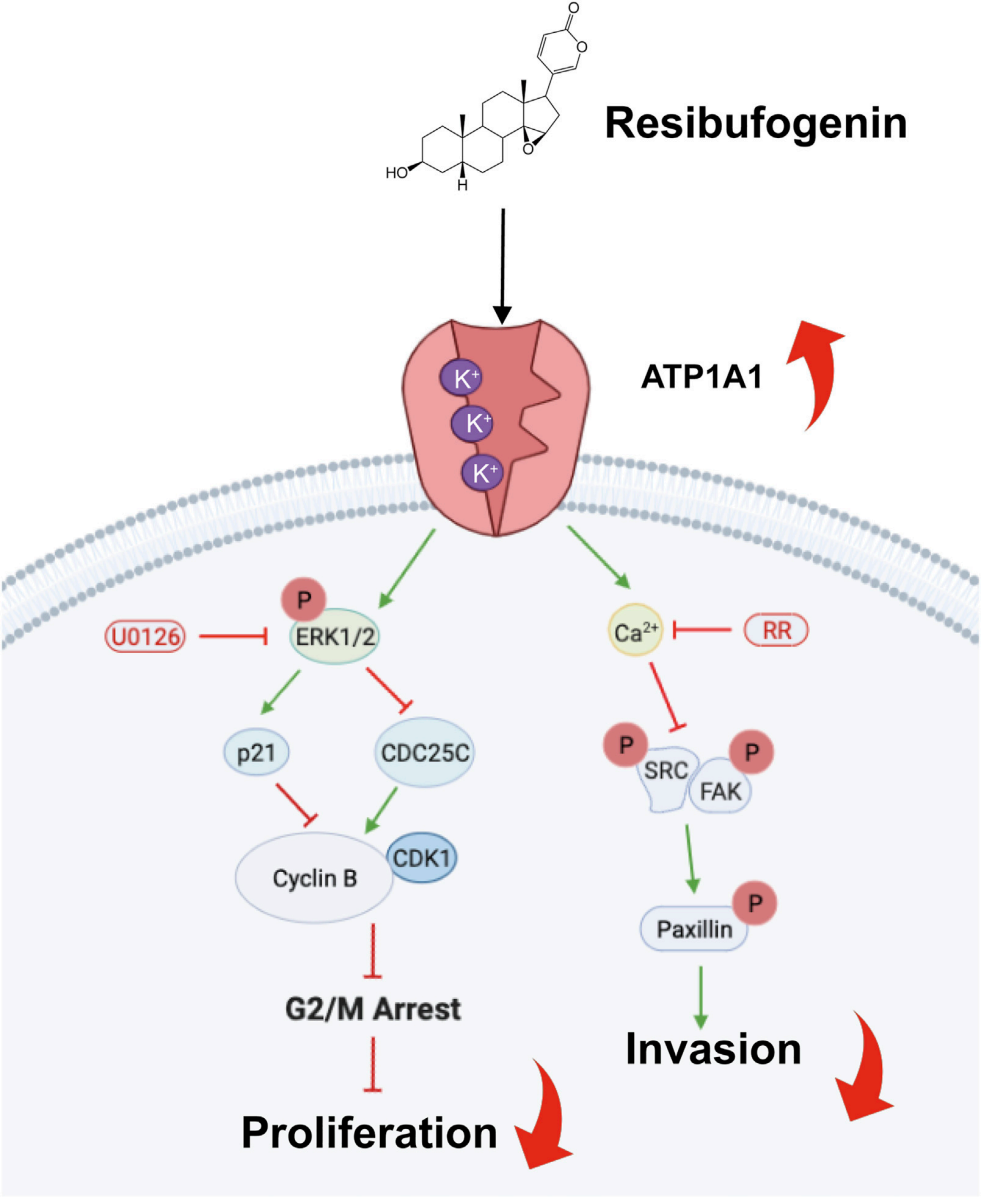
Scheme showing the central role of RB targeting ATP1A1 and inducing G2/M phase arrest and inhibiting cell invasion.

In recent years, the use of RB in cancer treatment has attracted increasing attention worldwide. Previous studies have shown that RB has anticancer properties. Intriguingly, we provided the first systematic analysis of the anticancer property of RB on glioblastoma *in vitro* and *in vivo*. This study demonstrated that RB binds to ATP1A1 and increases Na^+^-K^+^-ATPase activity, which activates the MAPK/ERK pathway, which regulates the expression of CDC25C and p21, followed by a reduction in the activity of the CDK1-Cyclin B complex, ultimately leading to G2/M phase arrest. Furthermore, the increase in Na^+^-K^+^-ATPase elevates intracellular Ca^2+^ accumulation to inhibit invasion by suppressing the Src/FAK/Paxillin focal adhesion pathway in GBM cells (Figure 7). This study not only contributes to research on RB in glioma but is also the first report to elucidate the target of RB in the inhibition of the malignant progression of GBM.

Chansu, known as a cardiac glycoside, has long been used in the treatment of disease. Digoxin, ouabain, and digitoxin are common cardiac drugs used to treat heart disease by blocking Na^+^-K^+^-ATPase and raising intracellular Ca^2+^ concentrations (Ahmed et al., 2008; Gjesdal, Feyzi and Olsson 2008). Additionally, these drugs were shown to be beneficial for patients with breast cancer (Stenkvist et al., 1979) and have been related to a reduced risk of lymphoma, leukemia, and urothelial and kidney cancers (Haux et al., 2001). Therefore, we further explored the antitumor potential of RB in glioma and identified a potential strategy for GBM therapy.

We found that at lower concentrations of 2 and 4 μM RB, Na^+^- K^+^-ATPase activity was activated. This result is in accordance with reports that the cardiotonic steroids ouabain, digoxin, and bufadienolides all have a reversed U-shaped dosage curve with suppression of Na^+^-K^+^-ATPase activity at higher doses and increased Na^+^-K^+^-ATPase activity at low doses (Bagrov, Shapiro and Fedorova 2009; Oselkin, Tian and Bergold 2010). We elucidated that 2 and 4 μM RB exhibited antitumor effects by activating Na^+^-K^+^-ATPase in cancer cells. In the future, we will explore the effects of wide-range concentrations of RB on glioma cell proliferation and invasion.

Inhibition of Na^+^- K^+^-ATPase generally leads to an increase in intracellular Na^+^ and a reduction in intracellular K^+^ and then induces an increase in intracellular Ca^2+^, which activates various pathways with a combination of genomic and nongenomic effects (Bagrov et al., 2009). However, in RB-treated glioma cells, we found that Ca^2+^ was significantly elevated after activation of Na^+^-K^+^-ATPase by RB, which inhibited the Src/FAK/Paxillin focal adhesion pathway. We hypothesize the existence of two possible mechanisms. A previous study hypothesized that Chansu induces the accumulation of intracellular Ca^2+^ possibly by acting at sites beyond Na^+^-K^+^-ATPase, either directly or indirectly through alteration in Ca^2+^ concentration (Bick et al., 2002). Another hypothesis is that activation of Na^+^-K^+^ ATPase by RB may stimulate the Na^+^- Ca^2+^ exchanger reverse mode, causing Ca^2+^ elevation. Na^+^-K^+^-ATPase is a special cell membrane protein that can breakdown ATP to obtain energy and use this energy for the active transport of Na^+^ and K^+^. The inactivation of Na^+^-K^+^-ATPase increases Na^+^ and attenuates Ca^2+^ extrusion through the Na^+^-Ca^2+^ exchanger, causing Ca^2+^ accumulation (Li et al., 2014). The mechanisms by which RB leads to the accumulation of Ca^2+^ in tumor cells deserve further study.

We observed a decrease in p-p38 in P3#GBM and A172 cells but not in U251 cells after treatment with RB. We thought that heterogeneity between GBM cells resulted in different phosphorylation levels of p38 after RB treatment. Therefore, the downregulation of p-p38 is not the key pathway of RB-induced GBM cell cycle arrest. We considered that the underlying reasons for the downregulation of p-p38 could be that 1) the inactivation of the FAK pathway induced by RB inhibiting the phosphorylation level of p38 (Alam et al., 2019) or 2) the decrease in intracellular ROS by RB-mediated activation of Na^+^-K^+^-ATPase leading to a downregulation in the phosphorylation of p38 (Maradagi et al., 2022).

This is the first preclinical study investigating the anticancer property of RB on GBM, and we investigated putative pathways induced by this compound. However, a limitation of this study is that the effects of RB in GBM were studied alone and not compared with those of temozolomide or the combination with temozolomide. Furthermore, Src/FAK/Paxillin focal adhesion pathways were detected via mRNA sequencing and Western blot assays. We need to further confirm that RB functions in GBM via Src/FAK/Paxillin focal adhesion pathways through well-designed experiments involving overexpression and knockdown of key components of these signaling pathways.

In summary, although our research on the functions of Chansu is far limited, the value of this class of compounds has raised increasing attention. These molecules play an important role not only in anesthesia and heart diseases but also in malignant tumors. Further investigation of these compounds will result in new and effective therapeutic strategies that are critical for optimizing health and treating disease. Further, our research demonstrates that RB inhibits GBM growth in tumor-bearing mice and prolongs animal survival. To expand the scope of the clinical application of RB, researchers must conduct large-scale, multicenter collaborative clinical trials in the future.

## Data Availability

The datasets presented in this study can be found in online repositories. The names of the repository/repositories and accession number(s) can be found in the article/Materials and Methods 2.11.

## Abbreviations

ANOVA: Analysis of variance; RB, Resibufogenin; RR: Ruthenium red; GBM: Glioblastoma; ROS: Reactive oxygen species; ATP1A1: Na^+^-K^+^-ATPase α1; WHO: World Health Organization; TAK1: Transforming growth factor beta-activated kinase 1; GSK-3: Glycogen synthase kinase-3; Tyr: Tyrosine; Ile: Isoleucine; Glu: Glutamic acid; Phe: Phenylalanine; Gly: Glycine; Thr: Threonine; Src: Nonreceptor tyrosine kinase; Ca^2+^: Calcium; Na^+^: Sodium; K^+^: Potassium; GSCs: Glioblastoma stem-like cells; MMP-2: Matrix metallopeptidase 2; JNK: C-Jun n-terminal kinase; DCFH-DA: 2′,7′-Dichlorodihydrofluorescein; KEGG: Kyoto Encyclopedia of Genes and Genomes; siRNA: Small interfering RNA.
