# Supplementary material for: Resibufogenin Targets the ATP1A1 Signaling Cascade to Induce G2/M Phase Arrest and Inhibit Invasion in Glioma
Source: Front Pharmacol. 2022 May 17;13:855626. doi: 10.3389/fphar.2022.855626 (PMC9152115; doi:10.3389/fphar.2022.855626)

Supplementary Material

Supplementary Figures

Supplementary Figure 1.

(A) For P3#GBM cells, we used RB concentrations of 0, 0.5, 1, 1.5, 2, 2.5, 3 and 3.5 μM for 48 h. For GBM cell lines U251 and A172, we used RB concentrations of 0, 1.5, 3, 4.5, 6, 7.5 and 9 μM for 48 h. For NHAs, we used RB concentrations of 0, 5, 15, 20, 25, 30, 35, 40 and 45 μM for 48 h. The IC_{50} values were 2.29 μM, 3.04 μM, 6.21 μM and 32.66 μM in P3#GBM, U251, A172 and NHA cells. **(B)** The population of P3#GBM, U251 and A172 GBM cells in the cell cycle phase after 48 h of exposure to vehicle, DMSO (0) or 2 or 4 μM RB was analyzed by flow cytometry. **(C)** P3#GBM and U251 cells were pretreated with 10 μM U0126 (ERK inhibitor) for 6 h, and then, 4 μM RB was incubated for another 48 h. Cells were assayed with propidium iodide staining and flow cytometric analysis. Data from three independent experiments are displayed as the mean ± SEM by log(inhibitor) vs. response - Variable slope (four parameters) **(A)**

Supplementary Figure 2.

(A) The intracellular Ca^{2+} level in U251 and A172 cells treated with 4 μM RB was detected by measuring the fluorescence intensity of the calcium indicator Fluo-4/AM with a fluorescence microscope. Scale bar, 100 μm. **(B)** Graphic representation of the relative Ca^{2+} level compared to the control shown in **(A)**. **(C)** Intracellular Ca^{2+} levels in U251 and A172 cells after treatment with a combination of 3 μM RR and 4 μM RB were detected by measuring the fluorescence intensity of the calcium indicator Fluo-4/AM with fluorescence microscopy. Scale bar, 100 μm. **(D)** Graphic representation of the relative Ca^{2+} level in each group shown in **(C)**. **(E)** Intracellular Ca^{2+} levels in U251 cells after treatment with a combination of 3 μM RR and 4 μM RB were detected by measuring the fluorescence intensity of the calcium indicator Fluo-4/AM with a flow cytometer. **(F)** Graphic representation of the mean fluorescence intensity ratio of Fluo-4/AM in each group shown in **(E)**. **(G)** The numbers of invasive cells were counted in three representative high-power fields per transwell plate. Scale bar, 100 μm. Data from three independent experiments are displayed as the mean ± SEM by unpaired Student's t test **(B)** or one-way ANOVA **(D, F)**. *, $P < 0.05$; **, $P < 0.01$ and ***, $P < 0.001$.

Supplementary Figure 3.

(A) Heatmap showing the mRNA analysis of peroxisome signaling. **(B)** The intracellular ROS in P3#GBM, U251 and A172 cells treated with RB was detected by measuring the fluorescence intensity of DCFH-DA with flow cytometry. **(C)** Graphic representation of the mean fluorescent intensity ratio of DCFH-DA indicating the relative level of ROS shown in **Supplementary Figure 3B**. **(D)** The population of cells in the cell cycle phase from the NC, RB, siATP1A1#3 and siATP1A1#3 + RB groups were analyzed by flow cytometry. **(E)** Intracellular Ca^{2+} levels from NC, RB, siATP1A1#3 and siATP1A1#3+RB cells were analyzed by flow cytometry. **(F)** The numbers of invasive cells were counted in three representative high-power fields per transwell plate in each group. Scale bar, 100 μm. Data from three independent experiments are displayed as the mean ± SEM by unpaired Student's t test **(C)** *, $P < 0.05$; **, $P < 0.01$ and ***, $P < 0.001$.

Supplementary Figure 1

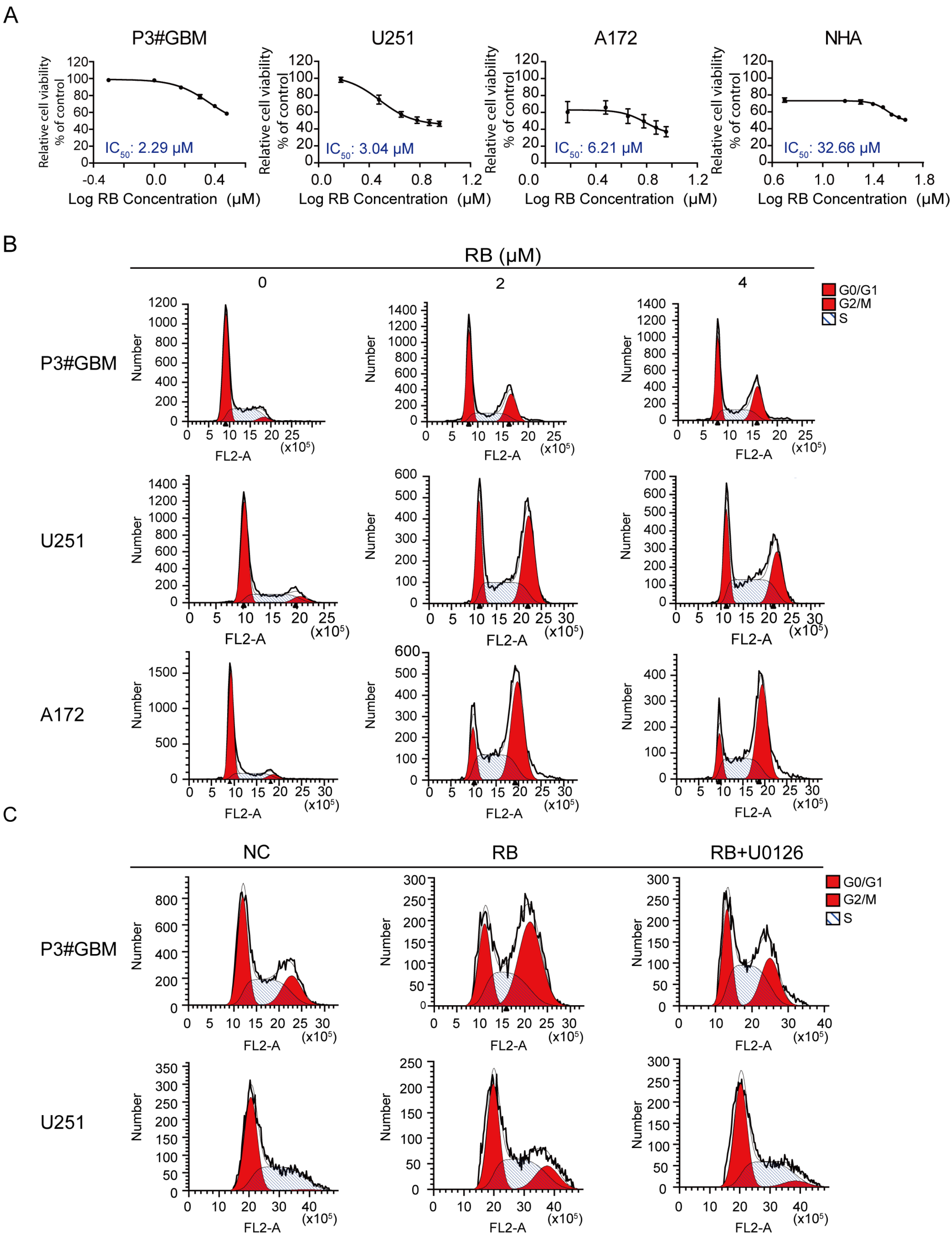

Supplementary Figure 2

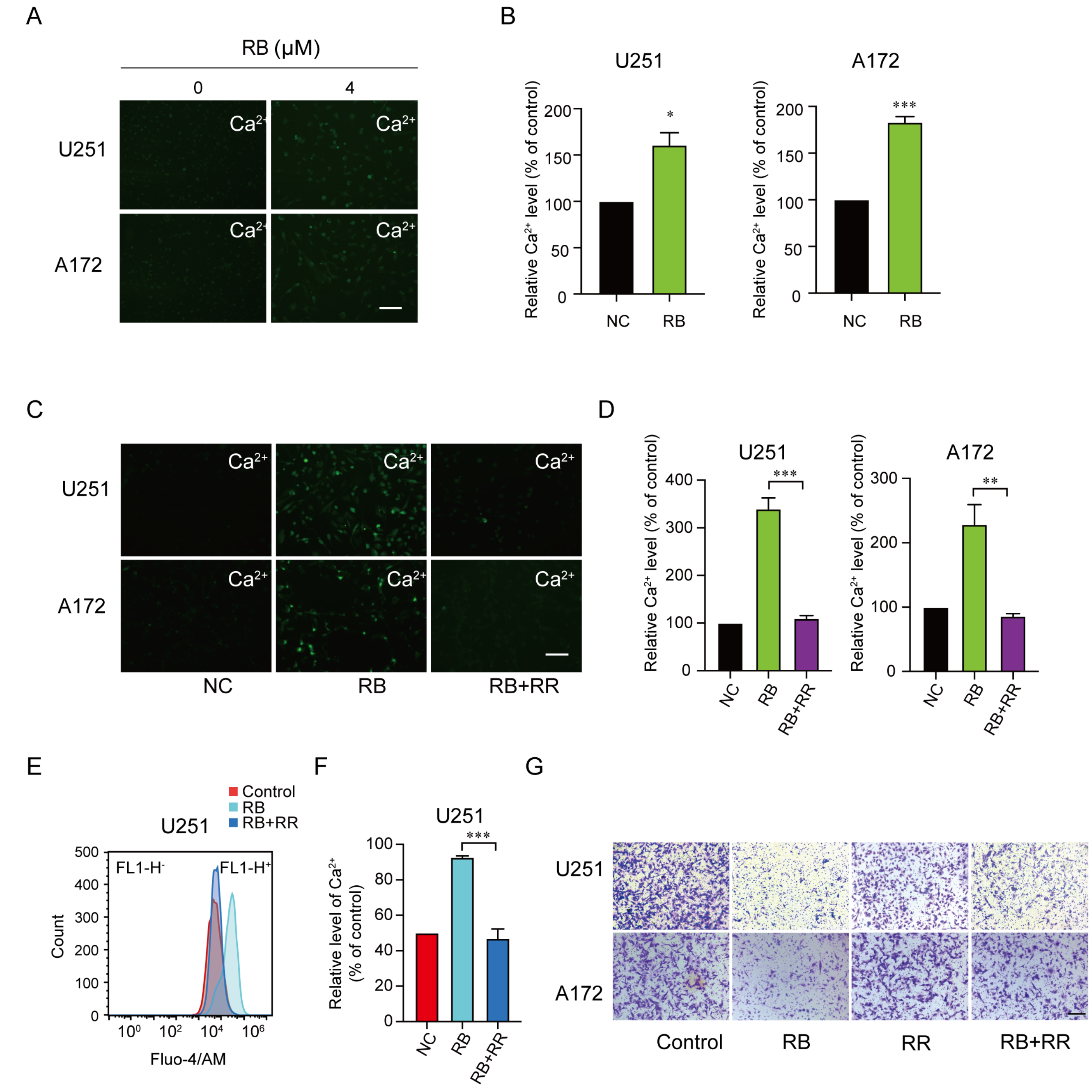

Supplementary Figure 3

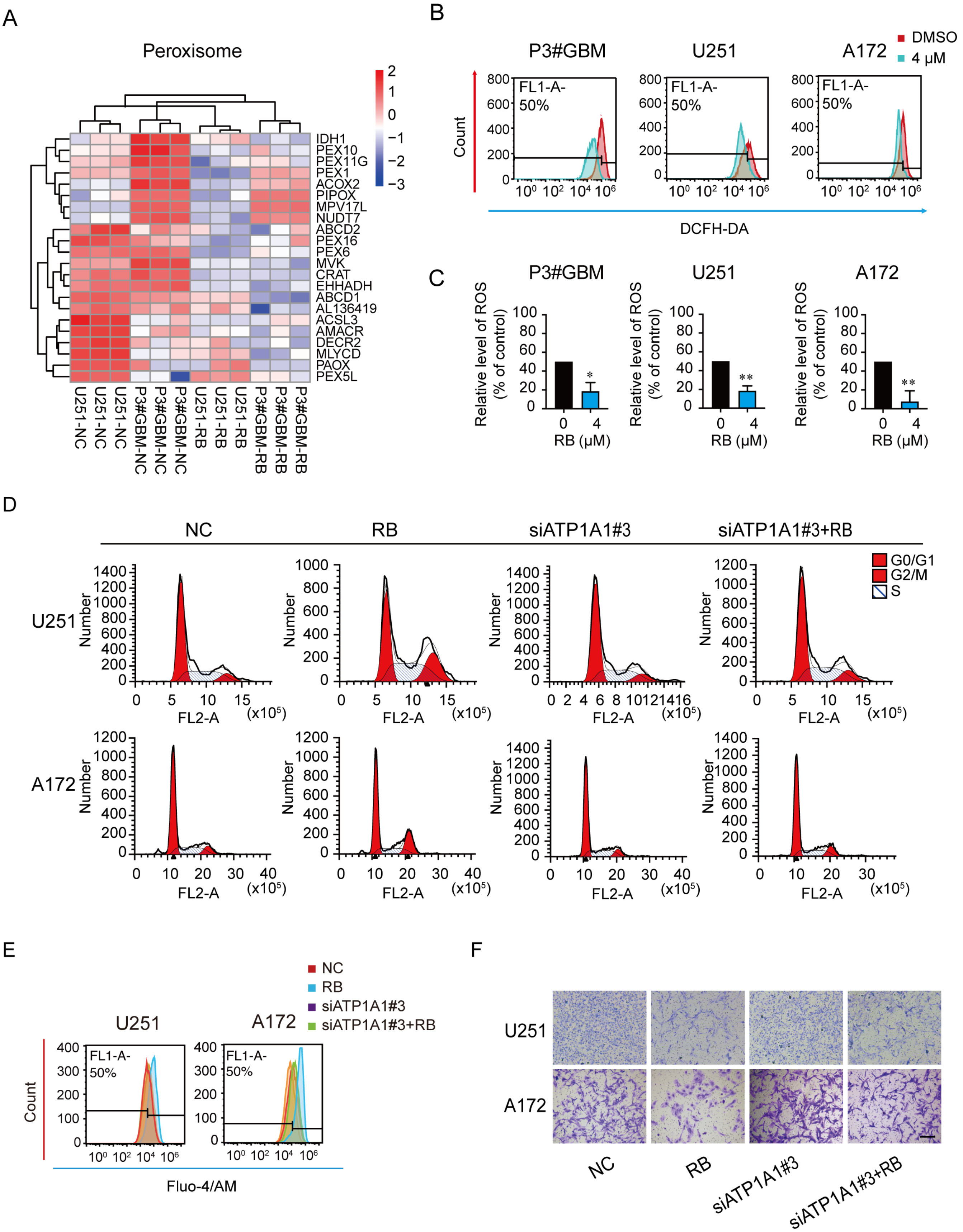

Supplement: Supplementary file 1 [file DataSheet1.pdf]
